# ADVANCEMENTS IN FRAILTY INTERVENTION RESEARCH: CURRENT TRENDS AND FUTURE DIRECTIONS

**DOI:** 10.1093/geroni/igae098.1275

**Published:** 2024-12-31

**Authors:** Qian-Li Xue, Qian-Li Xue

**Affiliations:** Chair; Discussant

## Abstract

Frailty, a complex, syndromic condition primarily affecting older adults, faces a lack of evidence-based management strategies due to scarce randomized controlled trials. So far, frailty interventions have employed a spectrum of strategies, including health behavior interventions (e.g., diet and exercise), pharmacological interventions targeting specific biological pathways, and personalized geriatric care models. However, the field faces significant challenges that hinder its progress. A notable issue is that there is a noticeable lack of comprehensive management strategies for frailty patients, supported by robust evidence. Moreover, trials examining the impact of physical activity and dietary interventions have largely focused on the clinical manifestations of frailty (e.g., strength) with little evidence of addressing its biological underpinnings. Further complicating these issues is the inconsistent validation of research findings across various studies and demographic groups, casting doubt on their broader relevance and efficacy. The symposium presents four talks: the first evaluates a values-based digital literacy intervention for prefrail and frail individuals with chronic disease; the next two discuss integrating technologies, including sensors with AI and voice-activated devices, for in-home frailty monitoring and intervention; the fourth considers mind-body practices’ (e.g., yoga and tai chi) potential to influence frailty’s biological underpinnings. Collectively, this symposium aims to: (a) differentiate between interventions tailored specifically to address frailty as a unique clinical entity and interventions aimed at improving care for frail older adults; (b) explore innovative approaches for tailoring frailty interventions to patient-specific goals; (c) tackle the inherent research and implementation challenges in frailty care.

